# Molecular Cloning and Functional Expression of the Equine K^+^ Channel K_V_11.1 (Ether à Go-Go-Related/KCNH2 Gene) and the Regulatory Subunit KCNE2 from Equine Myocardium

**DOI:** 10.1371/journal.pone.0138320

**Published:** 2015-09-16

**Authors:** Philip Juul Pedersen, Kirsten Brolin Thomsen, Emma Rie Olander, Frank Hauser, Maria de los Angeles Tejada, Kristian Lundgaard Poulsen, Soren Grubb, Rikke Buhl, Kirstine Calloe, Dan Arne Klaerke

**Affiliations:** 1 Department of Veterinary Clinical and Animal Science, Faculty of Health and Medical Sciences, University of Copenhagen, Frederiksberg C, Denmark; 2 Center for Functional and Comparative Insect Genomics, Department of Biology, Faculty of Science, University of Copenhagen, Copenhagen, Denmark; 3 Department of Large Animal Sciences, Faculty of Health and Medical Sciences, University of Copenhagen, Taastrup, Denmark; Universidad de La Laguna, SPAIN

## Abstract

The *KCNH2* and *KCNE2* genes encode the cardiac voltage-gated K^+^ channel K_V_11.1 and its auxiliary β subunit KCNE2. K_V_11.1 is critical for repolarization of the cardiac action potential. In humans, mutations or drug therapy affecting the K_V_11.1 channel are associated with prolongation of the QT intervals on the ECG and increased risk of ventricular tachyarrhythmia and sudden cardiac death—conditions known as congenital or acquired Long QT syndrome (LQTS), respectively. In horses, sudden, unexplained deaths are a well-known problem. We sequenced the cDNA of the *KCNH2* and *KCNE2* genes using RACE and conventional PCR on mRNA purified from equine myocardial tissue. Equine K_V_11.1 and KCNE2 cDNA had a high homology to human genes (93 and 88%, respectively). Equine and human K_V_11.1 and K_V_11.1/KCNE2 were expressed in *Xenopus laevis* oocytes and investigated by two-electrode voltage-clamp. Equine K_V_11.1 currents were larger compared to human K_V_11.1, and the voltage dependence of activation was shifted to more negative values with V_1/2_ = -14.2±1.1 mV and -17.3±0.7, respectively. The onset of inactivation was slower for equine K_V_11.1 compared to the human homolog. These differences in kinetics may account for the larger amplitude of the equine current. Furthermore, the equine K_V_11.1 channel was susceptible to pharmacological block with terfenadine. The physiological importance of K_V_11.1 was investigated in equine right ventricular wedge preparations. Terfenadine prolonged action potential duration and the effect was most pronounced at slow pacing. In conclusion, these findings indicate that horses could be disposed to both congenital and acquired LQTS.

## Introduction

In equine medicine, sudden deaths are a well-known problem and often the cause of death cannot be determined by necropsy [1]. Spontaneous arrhythmias have been shown to occur in horses [2], but studies coupling specific arrhythmias to sudden cardiac death (SCD) are sparse [3]. In humans, SCD in athletes has been linked to the Long QT Syndrome (LQTS) [4]. LQTS is characterized by a delayed repolarization of cardiac action potentials, a prolongation of the QT interval on the surface ECG and development of ventricular tachyarrhythmia of the *torsades de pointes*-type, which can progress to SCD. The presence of LQTS in veterinary patients has been suggested [5], however, reference values of the QT interval are not available for many companion animals and often the causes of unexpected deaths are not investigated in details [6].We have recently established the normal QT interval in standard and warmblood horses [7,8]

Repolarization of the cardiac action potential in larger mammals, including humans and horses, has been shown to be dependent on the rapid and slow activating delayed rectifier K^+^ currents, *I*_*Kr*_ and *I*_*Ks*_ [6,9,10]. Loss of function mutations in the genes encoding proteins mediating *I*_*Kr*_ or *I*_*Ks*_ are common causes of congenital LQTS in humans and pharmaceutical blockage of *I*_*Kr*_ is a well described cause of acquired LQTS. *I*_*Kr*_ is mediated by the pore-forming protein K_V_11.1 [11], which has been proposed to interact with the axillary β-subunit KCNE2 in cardiac cells [12]. K_V_11.1 is encoded by the *KCNH2* gene, also known as the *ether à go-go related gene* (ERG) due to similarities to the *Drosophila ether à go-go* (EAG) gene product [13]. The protein KCNE2 is encoded by the *KCNE2* gene [12]. Mutations in *KCNH2* have been linked to the long QT syndrome type 2 (LQT2) and mutations in *KCNE2* can give rise to LQT6 [12,14,15]. The K_V_11.1 channel is susceptible to pharmacological block by many compounds, including antiarrhythmic drugs as well as a variety of non-cardioactive drugs, which has been linked to acquired LQTS [16]. Acquired LQTS might also be of relevance to equine patients as horses are often treated with drugs known to prolong cardiac repolarization in other species, including quinidine, cisapride, erythromycin, and trimethoprim-sulfamethoxazole.

The aim of this study was to obtain a full length cDNA sequence of the equine *KCNH2* and *KCNE2* genes and to characterize the electrophysiological properties of equine K_V_11.1 and K_V_11.1/KCNE2 using the human isoform as reference. We found that the overall electrophysiological properties of the cloned equine channels are similar to those of the human isoform. However, equine K_V_11.1 currents were larger compared to the human homologue. We also found that *I*_*Kr*_ is physiologically important for cardiac repolarization in multicellular preparations of equine right ventricle. Our results elucidate the function of equine *I*_*Kr*_ in cardiac repolarization and indicate that congenital or acquired LQTS could potentially underlie unexpected deaths in horses.

## Materials and Methods

### Ethics Statement

Horses were donated to the Department of Veterinary Clinical and Animal Science, University of Copenhagen. The horses were euthanized by captive bolt gun and bleeding. No permit for animal testing was necessary under Danish law (The Animal Experimentation Act 1253 of 8^th^ of March 2013) to collect the equine tissue used for the experiments. In total, 7 horses were included, 3 geldings, 1 stallion 3 mares, age 9–10 years and of mixed breed. The prevalent reason for euthanasia was lameness and the horses had no history of any cardiac illnesses.

*Xenopus laevis* oocytes for heterologous expression were surgically removed from anesthetized frogs (anesthetic compound—0.2% 3-aminobenzoate methanesulfonate). The procedure was approved by the Danish animal experimentation board (approval number: 2012-15-2934-00625).

### PCR Primer Design

Predicted equine *KCNH2* and *KCNE2* gene sequences were obtained by using the Nucleotide Basic Local Alignment Search Tool (BLAST) [17] to compare the human *KCNH2* and *KCNE2* sequences (GenBank [18] accession numbers: NM_000218 and NM_172201.1, respectively) with the genomic sequence data from the EquCab2.0 *Equus caballus* genome project [19]. The predicted equine *KCNH2* and *KCNE2* sequences were used as the query sequence in BLAST covering all species with an annotated *KCNH2* and *KCNE2* gene. Following, portions with a high conservation were used as a basis for manual primer design. Primers were synthesized by Eurofins MWG Operon (Ebersberg, Germany).

### Bioinformatics

**Fig 1:**
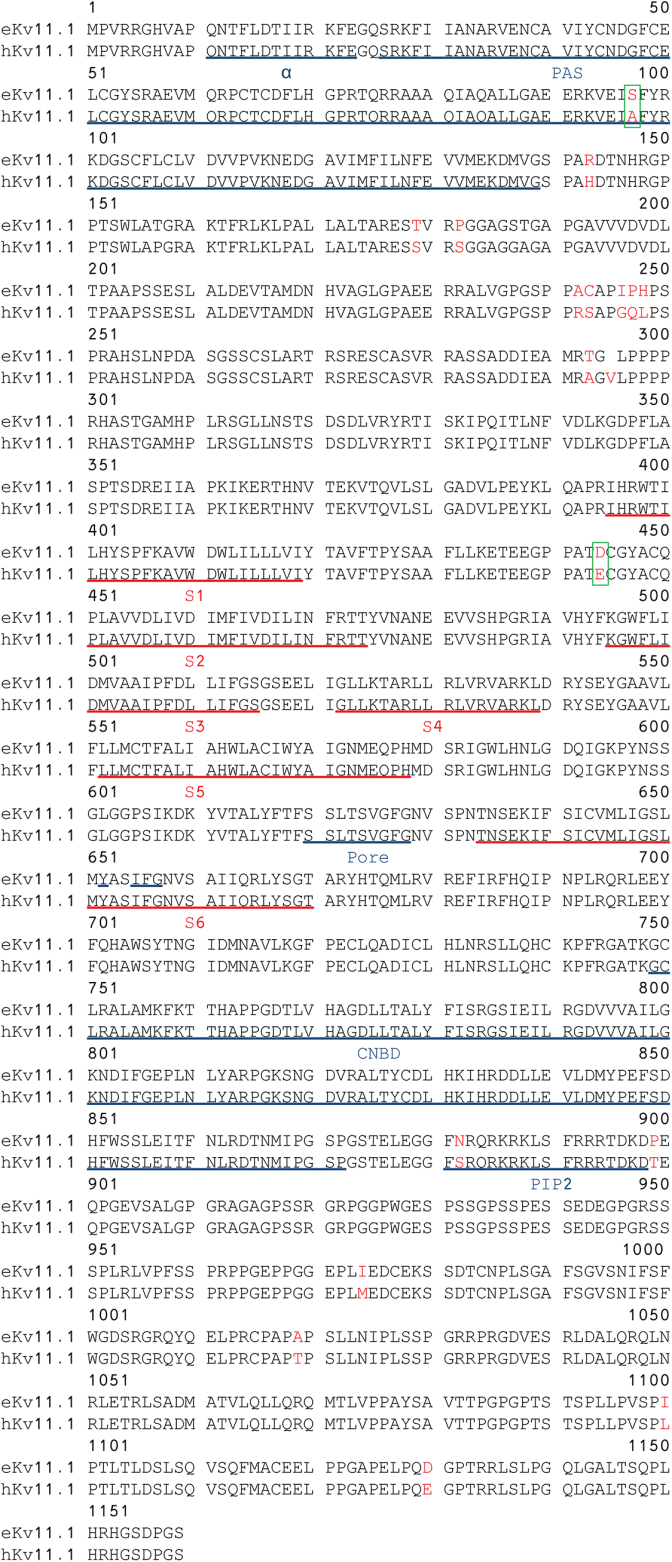
Alignment of human and equine K_V_11.1 protein sequences. Genbank accession number: Human NP_000229, horse ADK92992/ NP_001180587.1. The transmembrane domains S1-S6 are underlined in red. The α helix at residues 13–23, the PAS domain, the signature sequence at residues 620–629, the Y-652 and the IFG residues in S6, the cyclic nucleotide binding domain (CNBD) at residues 749–872 and the PIP2 binding domain are underlined in blue. Green boxes mark the equine amino acid in position A97S as this substitution in the PAS domain could be important for channel gating and position 444 as the E444D mutation has been published as a cause of long QT syndrome in humans.

**Fig 2:**
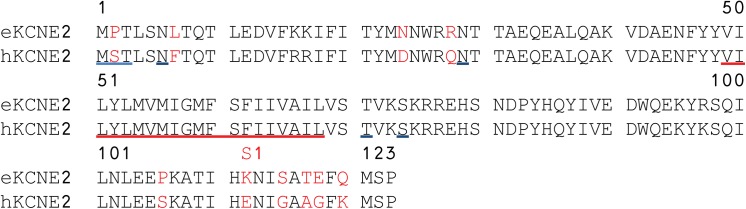
Alignment of equine and human KCNE2 protein sequences. Genbank accession number: Human NP_751951, horse AHH41329. The predicted three amino acids (MPT) initiating the equine KCNE2 protein sequence and the N6 and N29 glycosylation sites and the T71 and S74 phosphorylation sites are underlined in blue. The transmembrane region is underlined in red.

For further analysis of conservation, all species with an annotated *KCNH2* and *KCNE2* gene were found using the GenBank database or UniProt database [20] and these sequences were included in multiple sequence alignments generated using MAFFT [21] (Figs 1 and 2).

### Cloning of Full-length K_V_11.1 and KCNE2 cDNA

Equine myocardial tissue was sampled from the left ventricular free wall less than two minutes after euthanasia. Total RNA was isolated using Trizol reagent (Invitrogen). cDNA was synthesized using the SuperScript III First-Strand Synthesis SuperMix (Invitrogen) or, for Rapid Amplification of cDNA Ends (RACE), the FirstChoice RLM-RACE kit (Ambion) was used. Size of PCR products were confirmed by gel electrophoresis and subsequently cloned into the pCR4-TOPO vector (Invitrogen) using the TOPO TA cloning kit (Invitrogen). Following, the ligated vectors were transformed into One Shot Top10 and DH5α-T1^R^ competent cells (Invitrogen). Plasmid DNA was purified using the GenElute Plasmid Miniprep and kit (Sigma-Aldrich) and sequenced (Eurofins MWG Operon). Ligation of overlapping coding PCR products was done using T4 DNA ligase after cutting with relevant restriction enzymes (New England Biolabs). 5’ RACE PCR was unsuccessful for KCNE2. Based on our sequencing results from conventional PCR, the predicted equine *KCNE2* sequenced obtained from EquCab2.0 [19], and sequence alignment with *KCNH2* from other species, it was rationalized that the three amino acids (MPT) initiate the equine KCNE2 protein sequence at the amino terminus. Based on this assumption the predicted full equine KCNE2 was synthesized by GenScript (Piscataway). To facilitate expression in *Xenopus laevis* oocytes, the equine K_V_11.1 and KCNE2 cDNA was subcloned into the pXOOM expression vector [22] and sequenced (Eurofins MWG Operon). Human K_V_11.1 (NM_000218) and KCNE2 (NM_172201.1) in pXOOM were kind gifts from Dr. Thomas Jespersen.

### *In Vitro* Transcription of mRNA

Following linearization of the expression constructs with XbaI (New England Biolabs), mRNA was produced using the mMessage mMachine kit and purified with MEGAclear (Ambion). mRNA was stored at -80°C until use.

### Isolation of Oocytes and Injection of RNA for Heterologous Expression

Preparation of oocytes from *Xenopus laevis* was performed as previously described [23]. In brief, ovarian lobes were excised from the abdominal cavity of anaesthetized frogs. The ovarian lobes were digested with collagenase and incubated with hypertonic phosphate buffer to clear the follicle cell layer from the oocytes. Stage V or VI oocytes were selected and injected with approximately 50 nl mRNA solution (10 ng for K_V_11.1 alone and 10/20 ng for K_V_11.1/KCNE2) using a micro-injector (Nanoject, Drummond Broomall). Following, the oocytes were incubated approximately 48 hours at 19°C in kulori medium (in mM): 90 NaCl, 1 KCl, 1 MgCl_2_, 1 CaCl_2_, 5 HEPES, pH 7.4

### Two-Microelectrode Voltage Clamp of Oocytes

Currents were recorded by two-electrode voltage clamp using an Oocyte Clamp amplifier (OC-725 B, Warner Instruments) and a PC-interface (Axon Digidata 1440A, Molecular Devices). Data were sampled at 2 kHz using pClamp (Axon V. 10.2.14, Molecular Devices). Electrodes were pulled from capillary glass (TW 120–3, WPI) on a programmable micropipette puller (P-97, Sutter Instrument, Novato, CA, USA). Electrodes were filled with 1 M KCl and electrode resistance ranged from 0.5 to 1.5 MΩ. Experiments were performed at room temperature (19–21°C) in an air-conditioned room with the oocytes in a bath under a continuous flow of kulori medium. The experiments were repeated in at least three different batches of oocytes and qualitatively similar results were obtained.

**Fig 3:**
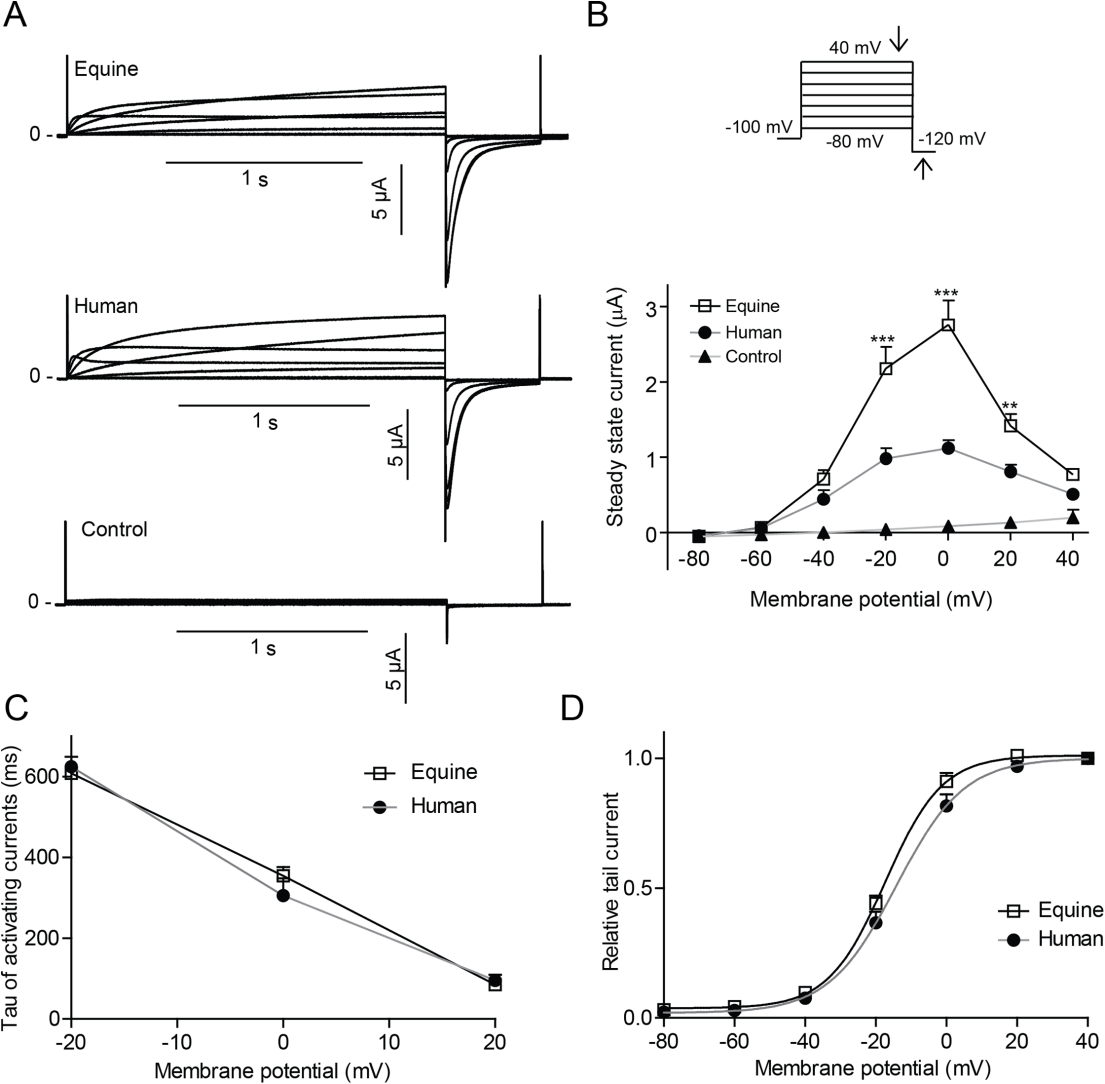
Equine and human K_V_11.1 expressed in *Xenopus laevis o*ocytes (A) Representative recordings of equine (n>14) and human (n>13) K_V_11.1 expressed in *Xenopus laevis* oocytes as well as uninjected controls (n = 15). (B) Steady-state currents (indicated by downward pointing arrow on the protocol) as a function of voltage. (C) Time-constants (Tau) of the activating currentss. (D) Peak tail currents (indicated by upward pointing arrow) normalized to maximal amplitude as a function of the voltage at the preceding step.

**Fig 4:**
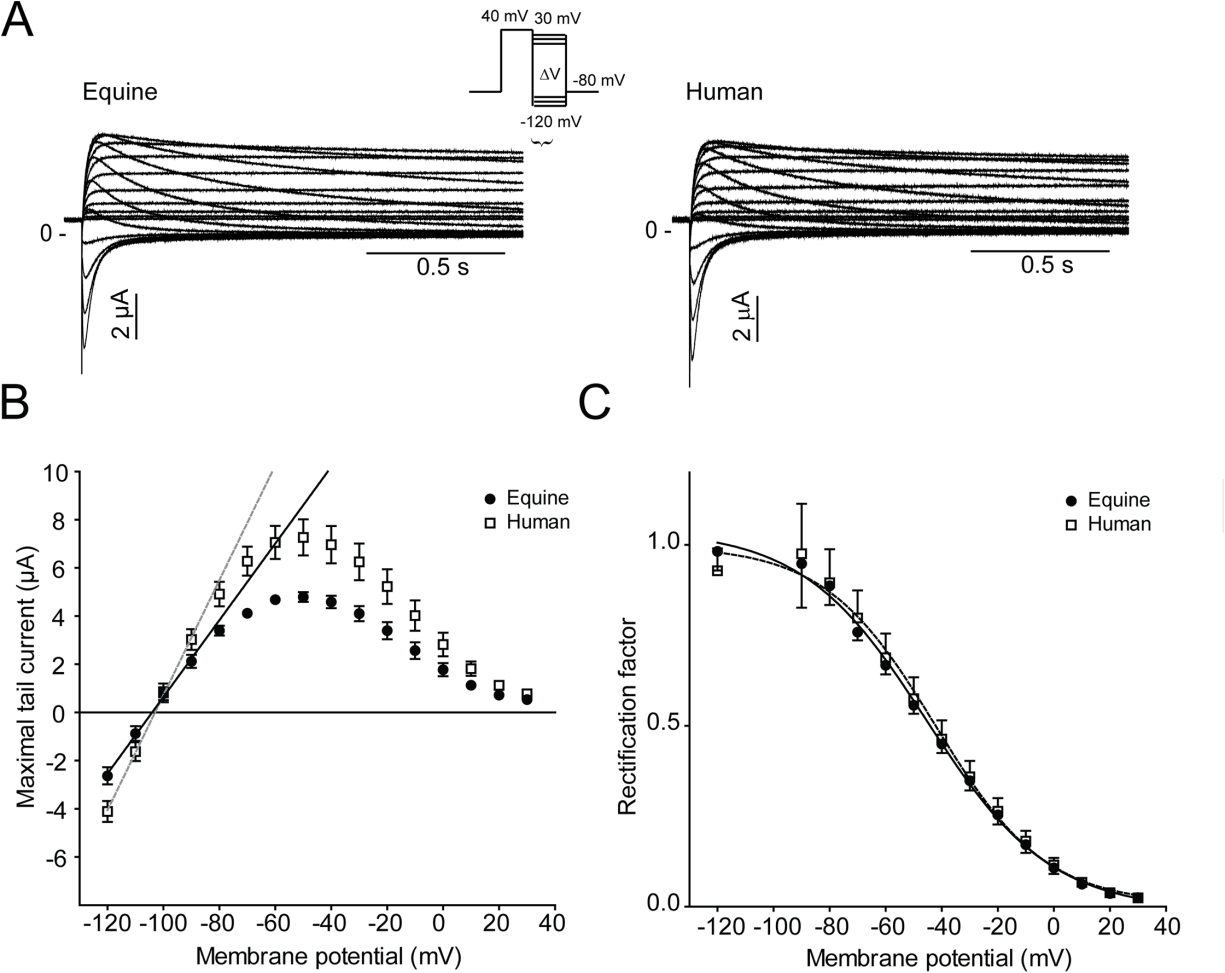
K_V_11.1 channel rectification and voltage dependence of inactivation. (A) Representative recordings of equine (n = 10) and human K_V_11.1 (n = 10) expressed in *Xenopus laevis* oocytes. (B) Fully activated current-voltage (*I-V*) relationship of the equine and human K_V_11.1 channels. The maximal conductance (*G*) of the tail currents was determined as the slope of a linear fit to maximal tail current amplitudes at potential between -120 to -90 mV. (C) Voltage dependence of rapid inactivation of equine and human K_v_11.1. The rectification factor (*R*) at each potential was calculated using the current amplitudes plotted in Panel (B) (see Methods for calculation). Data were fitted with a Boltzmann equation.

**Fig 5:**
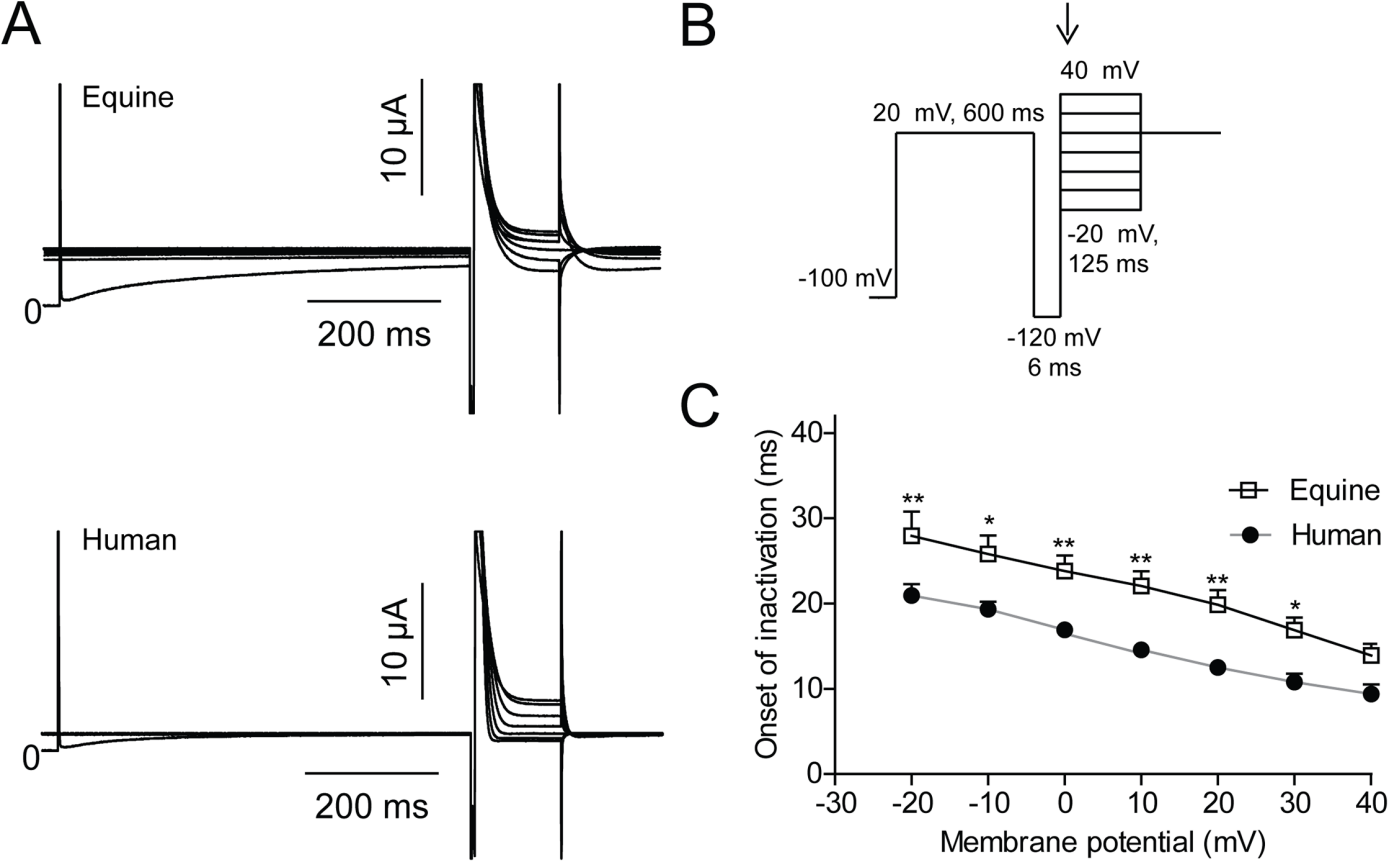
Time constants of onset of K_V_11.1 inactivation. Equine (n = 16) and human (n = 20) K_V_11.1 expressed in *Xenopus laevis* oocytes. (A) Representative recordings. (B) Voltage-clamp protocol. (C) Mono-exponential functions were fit to the inactivating currents as indicated by the arrow on the protocol and the obtained time constants were plotted as a function of voltage.

**Fig 6:**
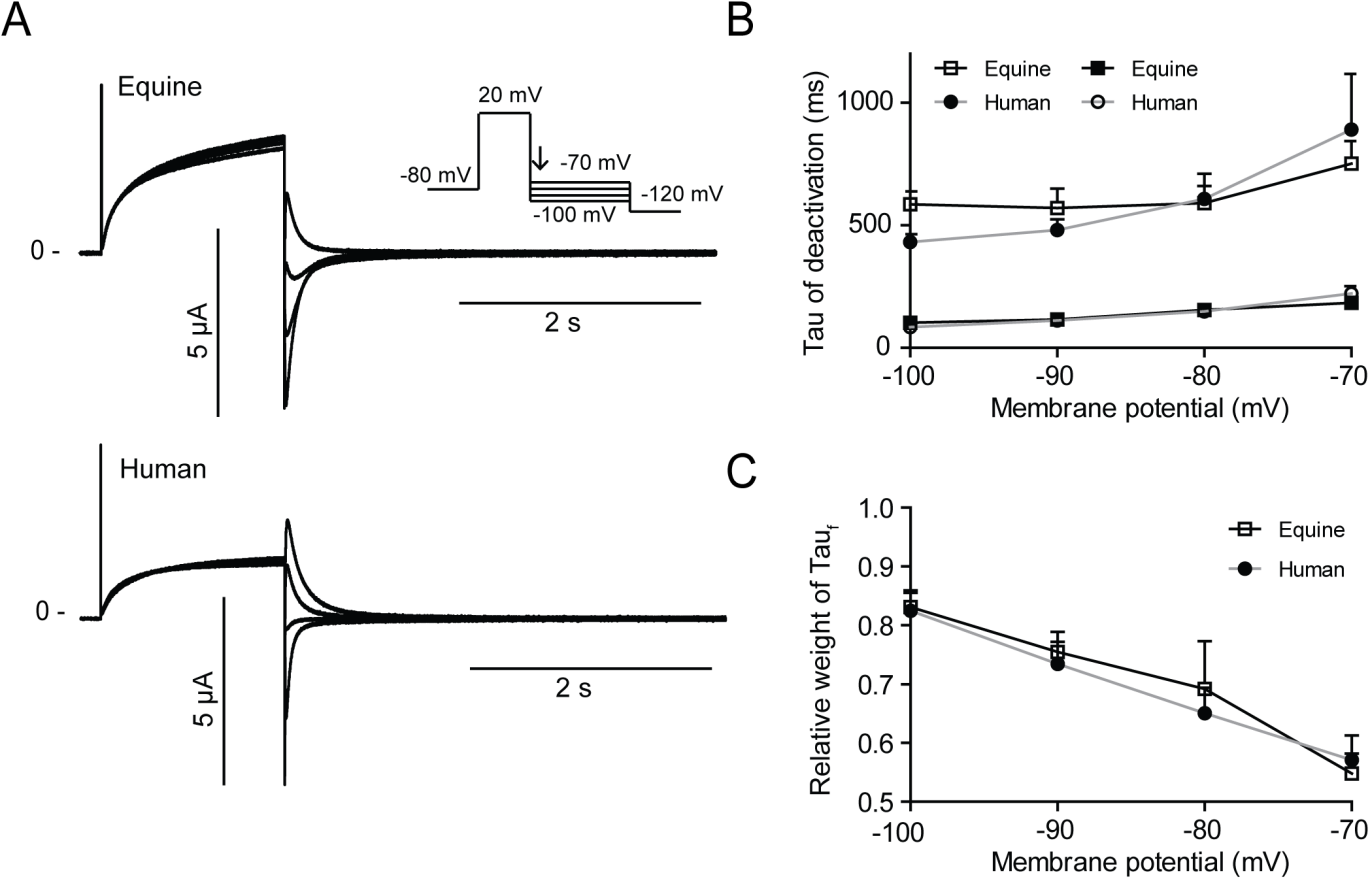
Time constants of K_V_11.1 deactivation. Equine (n = 25) and human (n = 25) K_V_11.1 expressed in *Xenopus laevis* oocytes. (A) Representative recordings. (B) Bi-exponential functions were fitted to the decaying currents (indicated on the protocol by an arrow) and the time constants τ_fast_ and τ_slow_ were plotted as a function of voltage. (C) The relative weight of the fast time constant (Tau_fast_).

Data were analyzed using Clampfit (Axon 10.4, Molecular Devices) and Prism 5 (GraphPad Software). The rate of activation of equine vs. human K_V_11.1 channel and K_V_11.1/KCNE2 channel complex was described by fitting a single exponential function *I*(t) = *A*_*i*_e^(-t/*τ*) + C to the initial 500 ms of the activating currents in Fig 3A. Only currents at -20 to 20 mV were included due to a prominent onset of inactivation at higher voltages. To address the voltage dependence of activation (Fig 3D), normalized peak tail current amplitude were plotted as a function of test potentials and a Boltzmann function, *I* = 1/(1+e^[(*V*_*1/2*_ *-V*_*t*_)/k]) was fitted to the data. *V*_*1/2*_ is the voltage required for half maximal activation of current, *V*_*t*_ is the test potential, and k is the slope factor. The rate of deactivation of equine vs. human K_V_11.1 channel and K_V_11.1/KCNE2 channel complex was obtained by fitting a double exponential function *I*(t) = ∑*A*e^(-t/*τ*) + C to the deactivating currents in Fig 4A. Voltage dependence of equine and human K_V_11.1 channel rectification from fast inactivation was determined by comparison of the fully activated *I-V* relationship for K_V_11.1 current with the *I-V* relationship expected for an Ohmic conductor (Fig 5A). A linear fit of current amplitudes between -110 mV and -90 mV describes the *I-V* relationship that would be found in the absence of rectification (Ohmic conduction). The slope determines the maximum conductance of K_V_11.1 tail currents, which can be used to calculate voltage dependence of channel rectification, as the rectification factor is given by *R = I*_*Kv11*.*1*_ / (G * n * (V_*t*_ *—E*_*Rev*_ *)* where *G* is the maximal conductance of K_V_11.1 tail currents, n is the activation variable at +40 mV (1.0), *V*_*t*_ is the test potential, and *E*_*rev*_, is the reversal potential. The rate of onset of inactivation was described by fitting a single exponential function to the data (Fig 6). To describe pharmacological block of the K_V_11.1 channel, terfenadine concentrations (0.01, 0.03, 0.1, 0.3, 1.0, 3.0, 10.0 μM) were transformed to log(concentrations) and plotted against current. A non-linear regression was fitted to the data, to obtain the IC_50_.

### Multicellular Right Ventricular (Wedge) Preparations

The right ventricle was excised and perfused with 4 x 50 mL heparinized (5 IE/L) cardioplegic solution (in mM: 129 NaCl, 12 KCl, 0.9 NaH_2_PO_4_, 20 NaHCO_3_, 1.8 CaCl_2_, 0.5 MgSO_4_, 5.5 glucose, pH 7.4, 4°C) through the coronary artery immediately after isolation and transported in 5 L of heparinized cardioplegic solution. Transmural wedges (4 x 3 x 2 cm) from the right ventricular wall were dissected, canulated and perfused arterially with cardioplegic solution. Leaks were ligated and the perfusion cannula was sutured to the wedge using 5–0 Mersilene suture (Ethicon GmbH). Only right ventricles were used as thickness of the left ventricle made it difficult to obtain viable preparations. The wedge was transferred to a tissue bath and perfused with oxygenated (95% O_2_ and 5% CO_2_) Tyrode’s solution (in mM: 129 NaCl, 4 KCl, 0.9 NaH_2_PO_4_, 20 NaHCO_3_, 1.8 CaCl_2_, 0.5 MgSO_4_, 5.5 glucose, pH 7.4) at 36±1°C and at a constant flow rate using Reglo Digital MS-4/8 tubing pump (Ismatic). The flow was set to 15–20 mL/min depending on wedge size. The wedge was paced from the endocardial surface using DS3 Constant Current Isolated Stimulator (Digitimer Ltd). Basic cycle lengths (BCLs) of 4000, 2000, 1000, 500, 333 and 250 ms were used. Recordings from the midmyocardium were made 30 min after cannulation (control) and 30 min after application of terfenadine (10 μM) using a floating microelectrode made from 1B100F-4 glass capillaries with filament (WPI). Microelectrodes were pulled on a Model P-97 Micropipette Puller (Sutter Instruments) to a resistance of ≈55 MΩ, when filled with 3 M KCl and connected to a Model 3100 Intracellular Electrometer (A-M Systems). A transmural ECG was recorded using Ag/AgCl half cells (Warner Instruments) mounted approximately 1 cm from the endo- and epicardial surfaces of the wedge and connected to an ISO-80 Isolated Bio-Amplifier (WPI). Data was recorded and digitized using PowerLab 4/20 (ADInstruments) and analyzed using LabChart v8.0.2 (ADInstruments). Chemicals other than enzymes and kits were obtained from Sigma-Aldrich.

### Statistics

All data are expressed as mean±SEM. Normal distribution was tested prior statistical analysis with KS test (Kolmogorov- Smirnov test with Dallal-Wilkinson Lillie for p-value) and all data was normal distributed except for Tau values describing K_V_11.1 deactivation, where the non-parametric Kruskal Walis and Man Whitney tests were used. Single outliers being more than three times the SD from the mean were removed from the dataset. Unpaired t- test was used to compare the equine and human half maximum activation V½ and the slope factors k. All other data were analyzed using two way ANOVA followed by a Bonferroni test. Statististical level of significance on figures are shown with *: p = ≤0.05, **: p = ≤0.01, ***: p = ≤0.001. All data was analyzed in Prism5 (GraphPad).

## Results

The full sequence of the equine K_V_11.1 and the partial sequence of the equine KCNE2 cDNA sequence were submitted to GenBank with the accession numbers HM641824 and KF937396, respectively. As the K_V_11.1 channel sequence is 3477 base pairs and highly GC rich it was difficult to amplify. Therefore, the full sequences was based on a full sequence from only one horse, which was verified by >50 partial sequences (50–500 base pairs, all with 100% homology) from five other horses covering different regions, but not the full sequence. Furthermore, our equine K_V_11.1 sequence have undergone validation (NP_001180587.1) by Genbank based on comparison to genomic sequence data (AAWR02041039.1) and the transcript is supported by transcript alignments and orthologous data. The submitted equine KCNE2 sequence corresponds to a predicted transcript (XP_001494244) based on computational analysis of a genomic sequence, using Gnomon (NW_001867397.1). The equine K_V_11.1 cDNA sequence had 93% similarity to the human cDNA and the encoded protein sequence had 99% similarity to the human (Fig 1). The equine K_V_11.1 cDNA sequence had 88% similarity to the human gene. The pore, the transmembrane domains S1-S6, and the cyclic nucleotide homology binding domain (CNBD) of equine K_V_11.1 were identical to human K_V_11.1. Most deviations were found in the N-terminus proximal to the Per-Arnt-Sim (PAS) domain. A single substitution was found within the PAS domain. The PAS domain is important for channel assembly and for the slow deactivation gating kinetics of the channel [24] and, interestingly, the p.E444D mutation has been published as a cause of long QT syndrome in humans [25]. The equine KCNE2 sequence had 90% similarity to the human KCNE2 (Fig 2). The transmembrane domains were identical in equine and human KCNE2 and the differences in sequence were found in the distal N- and C-termini.

The cloned equine K_V_11.1 was functionally expressed in *Xenopus laevis* oocytes and representative equine and human K_V_11.1 currents recorded by TEVC are shown in Fig 3A. Similarly to the human isoform, the steady-state current-voltage relationship was bell-shaped (Fig 3B), however, we repeatedly observed larger currents for equine K_V_11.1 compared to human (>6 different batches of *Xenopus laevis* oocytes, 3 different preparations of mRNA). Endogenous currents from control oocytes were insignificant (~0.2 μA) and did not affect measurements. As the proximal domain between the PAS and transmembrane segment 1 has been reported to be crucial for setting human Kv11.1 activation kinetics, time constants (Tau) of the activating currents were determined by fitting a mono-exponential equation to initial 500 ms of the current. No differences in Tau values were found (Fig 3C). It should be noted that since there is an overlap of the activation and inactivation processes both with regard to time- and voltage-dependence, the resulting Tau values reflect both these processes. The voltage dependence of activation for K_V_11.1 was addressed by plotting normalized currents measured at the -120 mV step as a function of the preceding test potential and a Boltzmann equation was fitted to the data (Fig 3D). Test pulses below -60 mV did not result in time-dependent currents. The half maximal activation (V_1/2_) was significant different (p = 0.0178) with V_1/2_ = -17.3±0.7, k = 8.1±0.7, n = 24 for equine and V_1/2_ = -14.2±1.1, k = 9.6±1.0, n = 16 for human. The slope factors K were not significantly different (p = 0.2143).

The degree of channel inactivation was determined from a fully activated K_V_11.1 current–voltage relationship (Fig 4). Currents were activated by a +40 mV step and peak tail currents were plotted as a function of potential (Fig 4B). The maximal conductance of the tail currents (*G*) was determined as the slope of a linear fit to maximal tail current amplitudes at potential between -120 and -90 mV (*G*_*equine*_ = 159±13 μS and *G*_*human*_ = 234±18 μS, n = 10). Voltage dependence of inactivation was estimated by the rectification factor *R* (Fig 4C). Reversal potentials were found to be *E*_*rev*, *equine*_ = -103 mV and *E*_*rev*, *human*_ = -104 mV, n = 10.

The speed of onset of inactivation was determined by activating K_V_11.1 currents by a +20 mV step followed by a brief step to -120 mV to release inactivation. A series of steps ranging from -20 mV to 40 mV resulted in a rapid inactivation of K_V_11.1 (Fig 5A). To obtain time constants, mono-exponential functions were fitted to the inactivating currents. The onset of inactivation was significantly slower for equine K_V_11.1 compared to human (Fig 5C).

Deactivation was addressed by activating channels by a +20 mV step and releasing inactivation by a series of hyperpolarizing steps from -100 to -70 mV (Fig 6A). Fast and slow time constants (Tau_fast_ and Tau_slow_) were found by fitting bi-exponential equations to the decaying currents and were similar for equine and human K_V_11.1 (Fig 6B). For both equine and human K_V_11.1, Tau_fast_ predominated over Tau_slow_ at more hyperpolarized voltages (Fig 6C).

**Fig 7:**
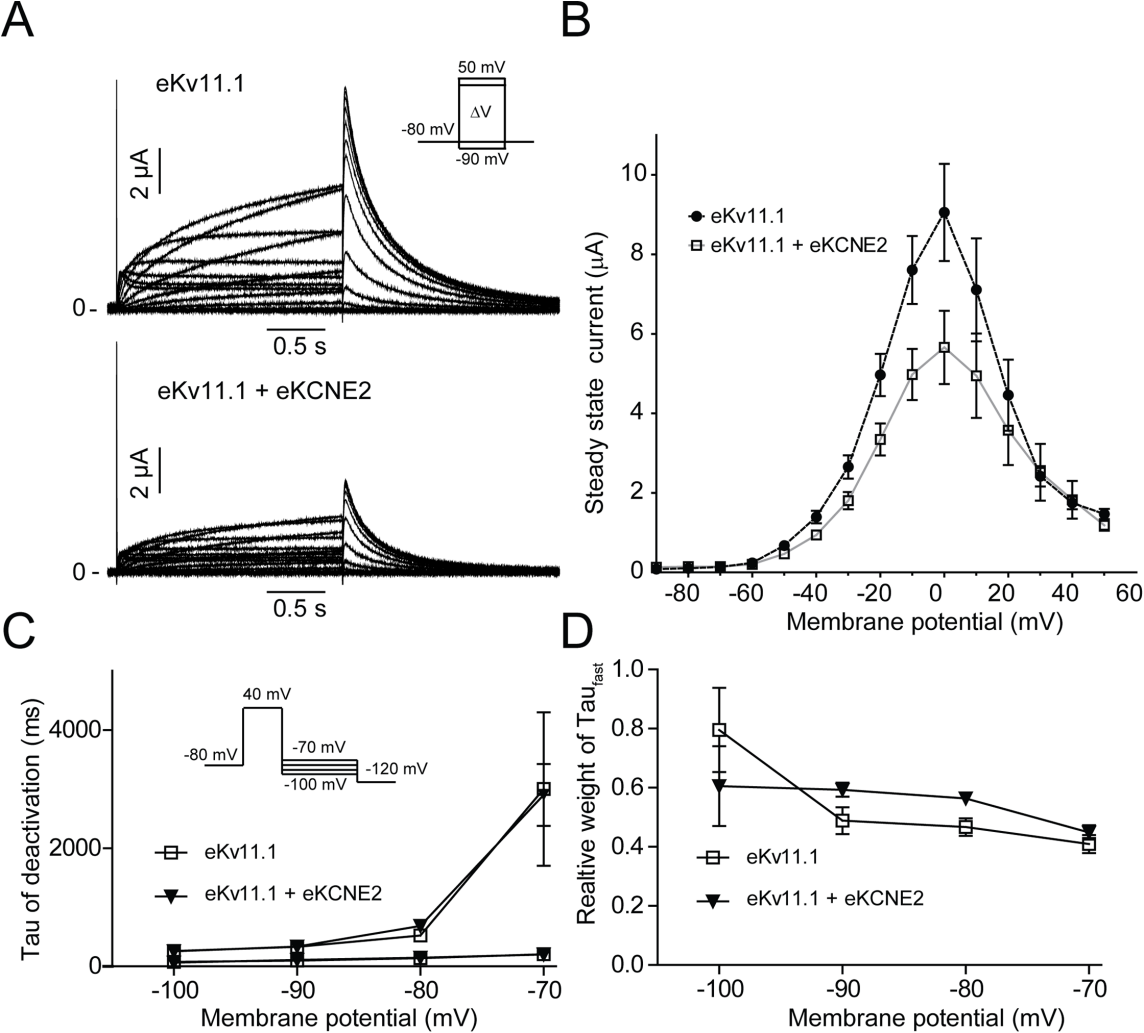
The effect of equine KCNE2 on equine K_V_11.1. Equine K_V_11.1 and K_V_11.1/KCNE2 expressed in *Xenopus laevis* oocytes. (A) Representative recordings. (B) Steady-state currents as a function of voltage, n = 10. C) Time constants (τ_fast_ and τ_slow_) of deactivation of equine K_V_11.1 (n = 8) and K_V_11.1/KCNE2 (n = 10) plotted as a function of voltage. D) The relative weight of the fast time constant (Tau_fast_).

K_V_11.1 and KCNE2 have been proposed to interact in cardiac cells [12]. Co-expression of equine K_V_11.1 and KCNE2 resulted in a reduction of the average steady-state current by 42.3±6.4% (Fig 7A and 7B). The gating properties were similar to those of homomeric K_V_11.1 channels (Fig 7A) and there were no effects on current deactivation (Fig 7C and 7D).

**Fig 8:**
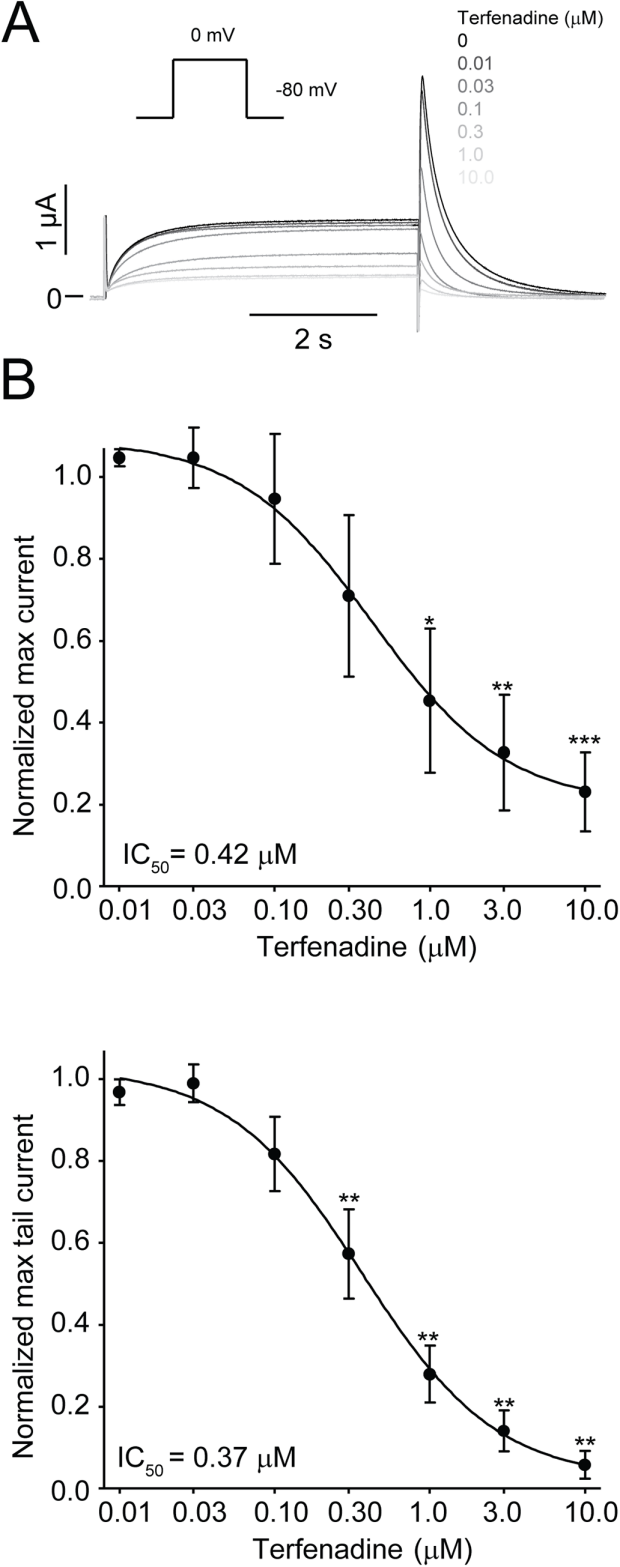
Equine K_V_11.1 channels are blocked by terfenadine. Equine (n = 4) K_V_11.1 expressed in *Xenopus laevis* oocytes. Currents were activated by a repeated depolarization to 0 mV from a holding of -80 mV. *(*A) Representative recordings in control and in the presence of 0.01, 0.03, 0.1, 0.3, 1, 3 and 10 μM terfenadine. Currents got successively smaller as concentrations were increased. (B) Dose-response for the effect of terfenadine on the equine K_V_11.1 steady-state currents at the end of a depolarizing step to 0 mV. (C) Dose-response for the effect of terfenadine on the equine K_V_11.1 peak tail current after repolarization from 0 mV to -80 mV. K_V_11.1 currents are expressed as a fractional value (*I*_*drug*_/*I*_*control*_). On the X-axis values non-transformed values are shown. A non-linear regression was fitted to the data.

Pharmaceutical block of K_V_11.1 can cause arrhythmias and sudden cardiac death [26]. Aromatic residues in the S6 transmembrane segment, Y-652 and F-656 are critical for the interaction with pharmaceutical compounds [27,28]. These residues are also present in equine K_V_11.1. Pharmacological block of the equine K_V_11.1 channel was tested with the histamine H1 receptor antagonist terfenadine. A concentration-dependent block of K_V_11.1 by terfenadine (0.01, 0.03, 0.1, 0.3, 1, 3 and 10 μM) was found (Fig 8). The IC_50_ was 0.42±0.14 μM for steady state currents and 0.37±0.06 μM for tail currents, n = 4.

**Fig 9:**
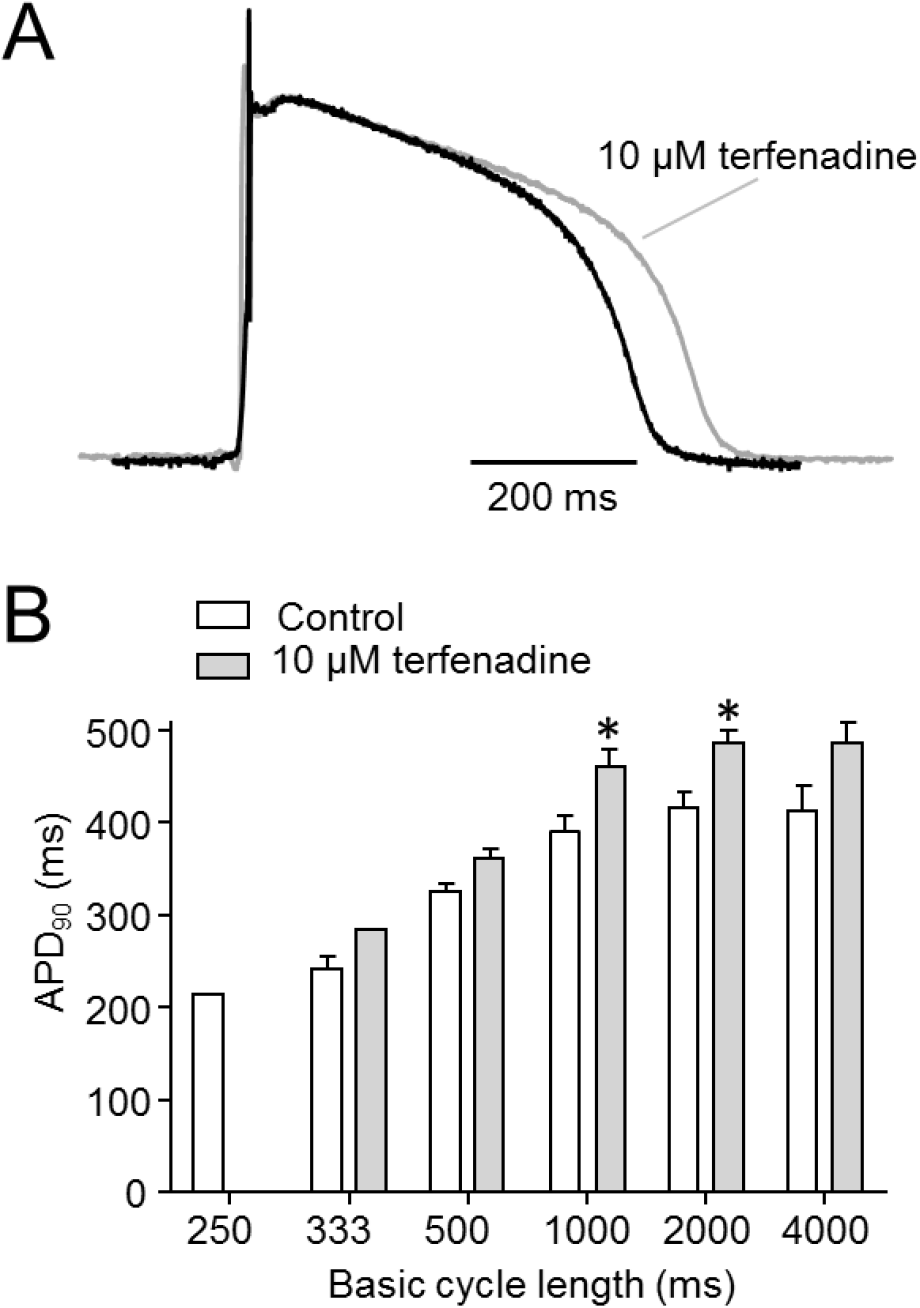
Physiological importance of KV11.1 in equine right ventricle. Action potentials were recorded from the midmyocardium in right ventricular wedges in absence or presence of terfenadine (10 μM). (A) Representative recordings at 2000 ms BCL. (B) Action potential duration at 90% repolarization (APD_90_) in absence or presence of terfenadine as a function of basic cycle length, n = 6.

To determine the physiological importance of K_V_11.1 currents in equine hearts, the effect of terfenadine (10 μM) was tested in arterially perfused sections of the right ventricle (RV), the wedge model. Action potentials were recorded from the midmyocardial region at different pacing rates (4000, 2000, 1000, 500, 333 and 250 ms BCL) using floating microelectrodes (Fig 9). In the presence of terfenadine, the action potential duration at 90% repolarization (APD_90_) was significantly increased at BCLs of 1000 and 2000 ms (Fig 9B). At 250 ms BCL we could not get capture in (5/6) wedges in controls, in the presence of terfenadine the prolonged action potential and concomitant increased refractory period prevented initiation of action potential at 250 ms BCL in 6/6 wedges.

## Discussion

In this study we successfully cloned full equine K_V_11.1 and partial KCNE2 cDNA. Currents were characterized by TEVC. The electrophysiological gating properties of the equine K_V_11.1 channel and K_V_11.1/KCNE2 channel complex were found to resemble those of the human K_V_11.1 and K_V_11.1/KCNE2 channels. The currents showed a bell-shaped steady-state current-voltage relationship that peaked at 0 mv due to a prominent inactivation. Furthermore, hyperpolarization resulted in a rapid recovery followed by slow channel deactivation. These electrophysiological properties are hallmarks of the K_V_11.1 channel and K_V_11.1/KCNE2 channel complex [12,29]. However, equine currents were larger compared to human K_V_11.1 and the voltage dependence of activation was shifted to more negative values. The onset of inactivation was slower in equine K_V_11.1 compared to human K_V_11.1, and we are speculating whether these differences in kinetics can contribute to the increase in K_V_11.1 current. There were no differences in the rectification of the K^+^ current, the voltage-dependence of inactivation, reversal potential or deactivation kinetics between equine and human K_V_11.1. Co-expression of KCNE2 resulted in a reduction of currents for both equine and human K_V_11.1. In accordance with Y-652 and F-656 being present in equine K_V_11.1, currents were blocked by terfenadine in the same concentration range as the human K_V_11.1 currents [28]. Finally we demonstrated that K_V_11.1 plays a functional role in cardiac repolarization in equine right ventricle, as application of terfenadine resulted in a prolongation of APD_90_ which was most prominent at slower pacing rates.

### Sequence and electrophysiological properties

The equine K_V_11.1 polypeptide sequence was highly similar to the human equivalent (99% similarity). This homology is greater than for any other previously sequenced animal K_V_11.1 polypeptide. Importantly, the signature sequence which shapes the selectivity filter of the channel was conserved. This part is essential to maintain the selectivity towards K^+^ and thereby the appropriate function of the channel [30] and in line with this, we found similar reversal potentials for equine and human K_V_11.1. Likewise, 100% conservation was seen for the transmembrane segments S1-S3, the S4 segment, which is essential for voltage sensing, and the pore forming S5 and S6 segments, which are hallmarks of voltage dependent K^+^ channels [31]. In the PAS domain, a p.A97S substitution was found in the equine K_V_11.1 polypeptide encoded by both the genomic sequence from the EquCab2.0 project [19] and the cDNA sequence obtained here.This substitution has not been reported in other species. Functionally the PAS domain is of importance for the deactivation kinetics [32], likely by interaction between residues 26–135 in the PAS-domain and residues 749–872 in the CNBD [33]. However, no differences in deactivation kinetics were found between equine and human K_V_11.1. A serine to alanine substitution is regarded as conserved suggesting that this substitution may be of little significance. To the authors’ knowledge, this mutation has not been published as a cause for altered properties of the K_V_11.1 channel. The CNBD domain was found to be 100% identical. Another important region of the K_V_11.1 channel is the amphipathic alpha helix at residues 13–23, which is involved in protein-protein and protein-membrane interactions [33]. This region was also found to be 100% identical in equine and human K_V_11.1isoforms. The majority of the 17 amino acid changes in the equine versus the human sequence map to the proximal domain between the PAS and the first transmembrane segment (S1). This region has been reported to be important for activation kinetics [34,35]. We found a left shift in the voltage dependence of current activation, however, the time constants of activation were similar for equine and human K_V_11.1. One interesting finding was the presence of a p.E444D substitution positioned in the extracellular loop between S1 and S2 in the equine K_V_11.1. This substitution is present in the peptide sequence of most species, except in humans and rabbits. p.E444D has previously been associated with long QT syndrome in a Chinese family [25], however, the electrophysiological properties of the substitution has not been tested *in vitro* [25] and the importance of the substitution remains speculative.

### Regulation of K_V_11.1 by KCNE2

The equine KCNE2 protein sequence was quite similar to the human equivalent (90% homology). The transmembrane regions were identical and the N6 and N29 glycosylation sites, as well as the T71 and S74 phosphorylation sites were conserved [36]. Co-expression with KCNE2 caused an approximately 40% reduction in steady-state equine K_V_11.1 currents, in agreement the effects of KCNE2 on human K_V_11.1 amplitude [12,37]. The reports describing the effects of KCNE2 on K_V_11.1 kinetics are not consistent (Reviewed in [36]). The role of KCNE2 in *I*_*Kr*_ has been questioned as co-expression of K_V_11.1 and KCNE2 does not recapitulate native *I*_*Kr*_ [38] and recently it has been proposed that KCNE2 is modulating K_V_11.1 by accelerating degradation of the K_V_11.1 protein [37]. It is, however, possible that interactions with other regulatory subunits affect the conductance and gating of the equine K_V_11.1 channel [32,39,40]. In humans, an association of the K_V_11.1 channel with the auxiliary β subunit KCNE1 has been proposed to regulate the K^+^ conduction of the channel [41] and in horses one study has described a possible interaction between K_V_11.1 and KCNE1 but no electrophysiological measurements have been performed on this equine channel complex [42]. Another reason for the discrepancy between native *I*_*Kr*_ and expressed K_V_11.1 could be the presence of different splice variants in native tissue. In humans, splice variants encoding a K_V_11.1 channel with a truncated N terminal (hERG1b) [43] and a c-terminal splice variant ERG_USO_ [40][44] has been reported. The ERG1b or the ERG_USO_ variants were not detected in horse hearts [42] but other splice variants could be speculated to be present.

### Pharmacology

The aromatic residues Y-652 and F-656 located in the central cavity of the K_V_11.1 channel have been demonstrated to be critical sites of interaction with structurally diverse drugs. Mutations of these residues have been shown to drastically decrease susceptibility of the K_V_11.1 channel to pharmacological block by various compounds [26,27].These residues were conserved in the equine K_V_11.1 sequence. To test if equine K_V_11.1 is equally sensitive to pharmacological blockade as the human isoform, we tested the effect of the histamine H1 receptor antagonist terfenadine. Terfenadine has been shown to block the human K_V_11.1 channel at concentrations relevant to its therapeutic levels [45], and it was removed from the market after frequent reports of syncope, QT prolongation and *torsade de pointes* in humans. The equine K_V_11.1 channel exhibited a dose-dependent block by terfenadine with an IC_50_ value of 0.42 μM, which is comparable to 0.36 μM described for the human K_V_11.1 channel [28].

### Physiological Importance of Equine K_V_11.1

The physiological importance of K_V_11.1 in equine hearts was determined by addition of terfenadine (10 μM) to arterially perfused wedges from the right ventricle. Terfenadine application resulted in a prolongation of the APD_90_ most prominently at slower pacing rates. At 2000 ms BCL, the APD_90_ was increased by 17% indicating that I_Kr_ plays an important role in action potential repolarization in equine right ventricle. These results are in agreement with Finley et al. that found a similar prolongation of APD_90_ after application of the *I*_*Kr*_ blocker cisapride to epicardial slice preparations of equine hearts [42]. Taken together, this suggests that LQTS, both in the congenital and acquired form could be of relevance in equine patients as in humans. In humans it has been proposed that a “repolarization reserve” exists [46]. Many different currents contribute to repolarization and as a consequence loss of one component (such as *I*_*Kr*_) normally does not result in repolarization failure, but if several components of the repolarization reserve are reduced, this may result in failure of repolarization [46]. It will thus be of great importance to determine other currents that are contributing to repolarization in equine cardiomyocytes.

## Conclusions

This study has shown that the equine K_v_11.1 and KCNE2 cDNA sequences are highly similar to the human and that they encode a functional K_V_11.1 and K_V_11.1/KCNE2 channel complex in *Xenopus laevis* oocytes. Equine currents were larger compared to human K_V_11.1 and the voltage dependence of activation was shifted to more negative values (V_1/2_ = -14.2±1.1 mV and -17.3±0.7, respectively). The onset of inactivation was slower in equine K_V_11.1 compared to human K_V_11.1. Furthermore, the equine channel is susceptible to the H1 receptor antagonist terfenadine in the same dose range as the human K_V_11.1 channel. Finally, application of terfenadine to a right ventricular wedge resulted in a prolongation of APD_90_, indicating that the K_V_11.1 channel plays a pivotal role in repolarization of the equine heart, as it does in the human heart.

The findings in this study suggest that horses could be disposed to both congenital LQT2, LQT6 and acquired LQTS. This is of importance to veterinary pathologists examining cases of sudden death in horses and to veterinary practitioners treating horses with drugs known to block the K_V_11.1 channel.

### Limitations

The experiments were performed at room temperature using *Xenopus laevis* oocytes. In *Xenopus laevis* oocytes, as in all heterologous expression systems, endogenous factors may affect the currents.
